# Exploring the valued outcomes of school-based speech-language therapy services: a sequential iterative design

**DOI:** 10.3389/fresc.2024.1290800

**Published:** 2024-01-19

**Authors:** Peter T. Cahill, Stella Ng, Lyn S. Turkstra, Mark A. Ferro, Wenonah N. Campbell

**Affiliations:** ^1^ School of Rehabilitation Science, McMaster University, Hamilton, ON, Canada; ^2^ Department of Speech-Language Pathology, University of Toronto, Toronto, ON, Canada; ^3^ Centre for Interprofessional Education, University of Toronto, Toronto, ON, Canada; ^4^ School of Public Health Sciences, University of Waterloo, Waterloo, ON, Canada; ^5^ CanChild Centre for Childhood Disability Research, Hamilton, ON, Canada

**Keywords:** outcomes, speech-language therapy, speech-language pathology, service delivery model, content analysis, mixed methods, structural topic modelling

## Abstract

**Background:**

Achieving outcomes that community members value is essential to high-quality, family-centred care. These valued outcomes should inform the production and interpretation of research evidence. To date, outcomes included in studies of service delivery models for speech-language services in schools have been narrowly defined, and do not match the outcomes suggested as important by families, teachers, and children. The most important outcomes of school-based, speech-languages services have not been directly and systematically investigated. We aimed to address this gap by asking school community members what outcomes were most relevant to evaluating and improving the delivery of speech-language services in schools.

**Methods:**

A sequential, iterative mixed-method study was conducted using interviews with 14 family members, educators, and speech-language therapists that asked what outcomes or impacts of school-based services they considered most important or valuable. Summative content analysis was used to analyse the data. Structural topic modelling between rounds of qualitative analysis was used to describe both the quality and the quantity of the interview content. School community members’ perspectives were compared through estimation of topic proportions within interviews from each member group and through qualitative comparison.

**Results:**

Structural topic modelling diagnostics and qualitative interpretation of topic output suggested a six-topic solution. This solution was estimated successfully and yielded the following topics: (1) meeting all needs appropriately, (2) teamwork and collaboration, (3) building capacities, (4) supporting individual student needs in context, (5) coordinating care, and finally (6) supporting core educational goals. Families focused on school-based services meeting all needs appropriately and coordinating care, while educators highlighted supporting individual student needs in context. By contrast, speech-language therapists emphasized building capacities and supporting core educational goals. All school community members agreed that current assessment tools and outcome measures were inadequate to capture the most important impacts of school-based services.

**Conclusions:**

Outcomes identified by school community members as important or valuable were broad, and included individual student outcomes, interpersonal outcomes, and systems-level outcomes. Although these outcomes were discussed by all member groups, each group focused on different outcomes in the interviews, suggesting differences in the prioritization of outcomes. We recommend building consensus regarding the most important outcomes for school-based speech-language services, as well as the prioritization of outcomes for measure development.

## 1 Introduction

Healthcare providers can improve family-centered care for children if they carefully and thoughtfully track and interpret meaningful outcomes (1–3). These outcomes include the results of care, the experiences that families have with their care and their satisfaction with the same, as well as the reduction or elimination of adverse events (3). A fundamental principal of family-centred care is the collaborative identification of desired service outcomes (4). Although clinicians offer important perspectives and knowledge, research indicates that there are important differences in values between practitioners and patients (5–7), with each contributing to shared, evidence-based decision making (8). Therefore, it is important to select core outcomes used to evaluate and improve health care through dialogue among all relevant parties.

Within paediatric speech language therapy (SLT), systematic reviews have highlighted important gaps in documented outcomes, including a paucity of participation-level outcomes (9, 10), as well as a lack of long-term outcomes and measures regarding family experiences with SLT services (10). Findings from qualitative research offer guidance regarding the kinds of outcomes that children and families might value. For example, Markham and colleagues (11) interviewed school-aged children with diverse speech, language, and communication needs regarding their quality of life. Qualitative analysis of these data suggested that children wanted positive social relationships, a sense of inclusion with family and peers, and a feeling of achievement and independence (11). Participants stated that they wanted to avoid being bullied, as well as feeling isolated or excluded (11). Lyons and Roulstone (12) also interviewed school-age children, this time with primary speech and language impairments, regarding their experiences in schools. These participants expressed their agency and independence, wanting to be recognized and included in their school environments, and resisted attempts of labelling, removal from the classroom, and separation from their peers (12). Similarly, these children identified difficulties with social relationships and challenges with academics as threats to their wellbeing, whereas agency and positive social relationships were supportive and protective of their health and happiness (13). Focus groups with parents from underserved areas of England (including parents of children receiving school-based services) also provided several suggestions regarding the improvement of services, including reduced wait times and increased time dedicated to clinician-family communication and rapport-building (14). Ethnographic research in schools has also suggested that parents want greater communication and care coordination to support their children with disabilities, including between health professionals working in schools and their children's educators (15). In summary, qualitative research suggests that children and families focus more on broader outcomes such as inclusion, wellbeing, and service quality than they do on children's specific skills and abilities.

Although these studies all provide windows into the perspectives of school-age children with communication disorders and their parents, few studies have explicitly and systematically asked multiple members of school communities about what they view as the desired outcomes of school-based SLT services (16). An exception is work by Gallagher and colleagues (17) that explored meaningful outcomes for children with developmental language disorder through focus groups with educators, parents, and clinicians and interviews with children. Using the qualitative data that emerged from the participation interactions in the focus groups, these researchers found that participants endorsed valuing the same broad outcomes, particularly academic and social participation, as well as self-management and advocacy (17). Nevertheless, there were important nuances among participant groups in how these broad outcomes were interpreted. For example, educators conceptualized academic participation primarily as the ability of children with developmental language disorder to participate in classroom activities and respond to teacher questions (17). Similarly, speech-language therapists (S-LTs) emphasized building the ability of children to identify when they were struggling with classroom language, and to know when to request assistance from teachers (17). By contrast, children emphasized being able to contribute meaningfully to classroom discussions and peer interactions, as well as navigating ethical dilemmas and complex social challenges with peers (17).

A clear opportunity remains to directly and systematically bring together diverse perspectives to identify the most valued outcomes of school-based SLT services. Although the work by Gallagher and colleagues (17) is a valuable contribution that directly addressed this issue, their findings were focused on children with a specific diagnostic label. In contrast, we wished to expand upon this previous work by exploring desired outcomes of school-based services for any child receiving or benefiting from SLT services in schools, including children without diagnostic labels. Additionally, we wanted to explore in greater detail desired outcomes within contemporary service approaches, such as tiered models that offer services across a continuum from universal, whole class to highly individualized (18). Prior research indicates that relevant outcomes in tiered service models may include student-, parent-, educator-, and systems-level outcomes, such as earlier identification of student needs, increased student participation in the classroom, expanded parent and educator capacities, fewer formal diagnoses, and reduced long-term burden of disabilities on the school community (19, 20). Interviews with S-LTs working in schools have confirmed that outcomes at these levels are relevant to practice and remain an area for professional growth (18). Consequently, it is timely to consider what outcomes of school-based SLT services are valued by members of school communities. Qualitative data provides a particular opportunity to explore the most valued outcomes of care, pivoting away from set questionnaires and ideas previously established in the literature, allowing instead participants with close knowledge of SLT services to describe their perspectives in their own words. Our research questions were as follows:

1. What outcomes are identified as valued or meaningful to family members, educators, and clinicians involved in school-based, SLT services?
2. What differences in these community members’ perspectives are reflected in the quality or quantity of their discussion of these outcomes?

## 2 Methods

In the present study, we explore meaningful outcomes for school-based services through a mixed-methods summative content analysis using interview data. Summative content analysis makes use of both qualitative and quantitative aspects of textual data to explore the usage and meaning of participants’ words (21). This approach is consistent with mixed methods assumptions that reject a strict duality between qualitative and quantitative data, and instead posit that data can be either qualitative or quantitative depending on how the researcher approaches the data (22). In this study, we represented the data both quantitatively (the frequency and co-occurrence of words), as well as qualitatively (interpretation of meaning via close reading by the researcher). We used a sequential iterative design (22), allowing the qualitative and quantitative analyses to mutually inform and develop the results.

### 2.1 Ethics

Study methods followed ethical guidelines and regulations. All materials and procedures for this study were reviewed by the Hamilton Integrated Regional Ethics Board (Project number #13906) affiliated with McMaster University, as well as the ethics committees of all participating school boards. All participants provided informed consent prior to initiating any study activities.

### 2.2 Sampling strategy

We used purposeful sampling (23), initially identifying interested and motivated S-LTs who would likely have rich perspectives on the research topic. Subsequently, we used snowball sampling (24), asking recruited participants to identify educators likely to have relevant knowledge and perspectives. This combined sampling approach has been recommended when attempting to elicit perspectives on a complex topic from the perspective of multiple member groups (25, 26). To recruit parents and caregivers, we reached out through known channels, harnessing the networks of research and clinical colleagues based at McMaster University's CanChild Centre for Childhood Disability Research. We used the concept of information power (27) to inform the final sample size, using our prior knowledge to set an *a priori* sample size and revising the same based on the variability of data collected. In this case, we originally planned on interviewing 20 participants; however, we reduced this number as the interviews rapidly reinforced the ideas from previous interviews as well as from prior work in this area [see (18)].

### 2.3 Participants

We recruited participants belonging to three school community member groups who we anticipated would have an interest in outcomes for school-based SLT services: families of children receiving these services (*n* = 4), S-LTs (*n* = 5), and educators (*n* = 5). All participants were connected to school boards (a term for a local educational authority) in Ontario, Canada, with the professionals employed directly by the school boards rather than by third party health agencies.

### 2.4 Materials and procedures

Interviews followed a semi-structured format. A common prompt was used to open every session, with prompts prepared for contingent response to the discussion. These prompts were used to follow up on ideas brought up by participants in response to the initial common prompt. Prompts were developed based on previous literature regarding outcomes for SLT services in schools (19) and school-based tiered services (20). See Additional file 1 for a copy of the interview guide. One pair of S-LTs preferred to be interviewed together, and so a simultaneous interview was conducted for these participants.

All sessions were conducted using videoconferencing software and were recorded with automated transcripts. Following each, the first author listened to the recording three times and corrected the transcripts. The transcripts were simultaneously de-identified with all names and other identifying references removed and replaced with non-identifiable placeholders. Corrected and de-identified transcripts were then uploaded to relevant data analysis software (see next section).

Finally, we used qualitative surveys subsequent to the interviews to collect additional data. These surveys provided an opportunity to further develop and expand on ideas explored in the original qualitative data collection (28). A link to these surveys was sent out to participants approximately one week following the interviews and all data was collected using Research Electronic Data Capture [REDCap: (29)].

### 2.5 Data analysis

#### 2.5.1 Data familiarization

We performed a summative content analysis (21) using data from the interviews. The analysis occurred in three steps. In the first step, the first author read all transcripts in their entirety to make sense of the data as a whole (30). Memo writing was used at this stage, recording initial questions and impressions of the data, and these initial impressions were discussed within peer debriefing between the first and last authors.

#### 2.5.2 Structural topic modeling

In the second stage, a quantitative analysis was performed. We used a topic modelling approach embedded within this summative content analysis, as computer-aided content categorization and counting is consistent with the paradigmatic assumptions of summative content analysis (31). All data were uploaded to R (32) software. Subsequently, structural topic modeling [STM; (33, 34)] was performed using the *stm* package (35). STM is a multi-class membership machine learning algorithm used to analyze textual data and their metadata (36). This algorithm searches through text calculating the frequency and co-occurrence of words to identify latent topics that are present in the data set (36), and to identify the terms most likely to belong to each topic.

##### 2.5.2.1 Data cleaning

We first cleaned the data for analysis. This process removes words and morphemes that provide little content information (37), such as articles (e.g., “the,” “a”) and most inflectional and some derivational morphology (e.g., “assessments” is reduced to “assess-” with “-ment-” and “-s” removed). This approach reduces the number of comparisons required by the algorithm and avoids cluttering the results with function words that provide little semantic information (37). To do so, we used the built-in lists with the *stm* package, and added additional conversational words, as the built-in lists were developed for use with formal written texts, as well as words unique to specific participants contexts (e.g., terms only used by their local educational authority).

##### 2.5.2.2 Model selection

We then applied STM to the data and used our understanding of the data from the original qualitative exploration of the data, as well as relevant previous literature, to interpret topics and inform the final selection of the number of topics to be retained in the model. We used goodness of fit statistics to guide the range of ideal topic numbers; however, we retained the primacy of the qualitative interpretation to select the final algorithm solution. We focused on the fit statistics of semantic coherence and exclusivity. These fit statistics are compared in relative terms to other topic number solutions for the same data set, rather than by reference to absolute cut-offs or reference values. Semantic coherence provides an estimate of how frequently words within the topic co-occur (35, 36), and is strongly associated with human judgement of topic coherence (38). Exclusivity opposes semantic coherence, and prefers topics structures where words are not shared among multiple topics (35, 36). Better fitting models can be identified through model solutions that optimize the values of these two opposing fit statistics (35, 36). The topics were then named based on qualitative interpretation of the top terms within each topic.

##### 2.5.2.3 Use of metadata

An advantage of STM for this project is that it does not suppose independence of the data and the data generating mechanism (36, 39). Consequently, the method allows a description of the differences in topic proportions across documents (36, 37). We postulated that different school community members may discuss different topics. This metadata would allow exploration of topic distribution among member groups. For each topic, we estimated the topic proportion differences across member groups to compare the quantity of data dedicated to each outcome.

#### 2.5.3 Qualitative interpretation and categorization

In the third step, topics from the final STM model were interpreted qualitatively by the research team using notes and memos from step 1 to help interpret the topics. The first author named the topics drawing on both the results of the quantitative model and qualitative familiarity with the data. The first author then reviewed the transcripts again with the topic solution in mind and selected emblematic quotes for each topic that illustrated the meaning and nuance of community members’ discussion of each outcome topic. Finally, the quality and quantity of the data were interpreted in light of both quantitative and qualitative results, as well as previous literature in this research domain. Peer debriefing between the first and last author was used throughout this step.

### 2.6 Legitimizing inferences

In mixed methods studies, researchers must develop and bolster high quality inferences (40). Inferences are the conclusions and interpretations of the research results (40). Achieving high quality inferences is a process that occurs throughout the entire research process, and is central to rigorous mixed methods research design (40, 41). This process has been referred to as *legitimation* (41), and can be considered analogous to validity and creditability in quantitative and qualitative paradigms, respectively (40).

To legitimize our inferences, we used several strategies. In keeping with recommendations for content analysis (30), we used peer debriefing regularly throughout the project, including between each phase of the analysis. This was necessary to explore perceptions and interpretations of the data up to that point, allowing the analysis to benefit and develop from multiple perspectives throughout the analytic process. Memo writing also was used regularly to document and enhance the analysis. Critical to this analysis, we used data analysis triangulation, using both qualitative and quantitative analysis techniques to generate and mutually inform the results. We used this data analysis triangulation as a form of weakness minimization (41), relying on qualitative reading and coding of the data to bolster inferences about the *quality* of the content, while using STM to bolster inferences about the relative *quantity* of topics and their distribution across the data set. Finally, we used both a close, human reading of topic content supplemented by a machine reading of topic quantity to make inferences from our text data (39). This approach maximized the amount of information available to the research team when generating inferences from the data.

## 3 Results

### 3.1 Step 1. Data familiarization

Initial qualitative impressions indicated that participants frequently focused on processes related to key outcomes (e.g., I must *collaborate with the teacher* in order to *achieve student progress*). Additionally, all participants appeared to generally agree that all outcomes were important, although the prioritization of each outcome may have differed among the member groups, as families particularly appeared to focus more on access to services and the provision of all appropriate services to students, whereas S-LTs and teachers focused more on collaboration and implementation in the classroom. Participants also appeared to discuss student-level, interpersonal, and systems-level outcomes as important and interrelated.

### 3.2 Step 2. Structural topic modeling

**Figure 1:**
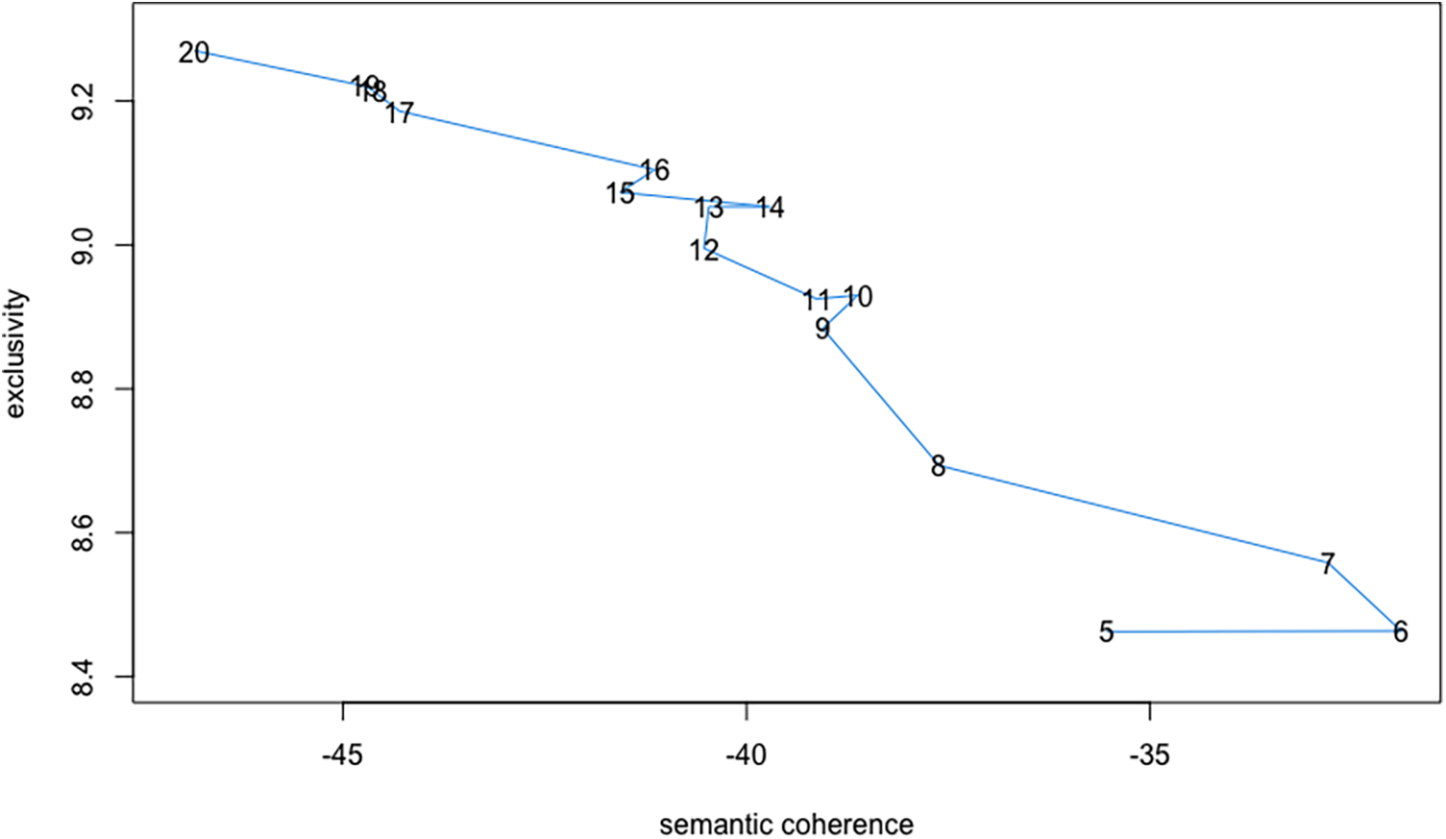
Semantic coherence and exclusivity per topic model.

We fit topic models to the transcript data. Only three follow up surveys were completed with very brief responses that reiterated discussion points in the interviews. As topic modelling can perform poorly on short text excerpts (42), we choose to exclude this data from the analysis. We started with a five-topic solution and proceeding until a 20-topic solution and then evaluated diagnostics, focusing on estimates of semantic coherence and exclusivity for each model. See Figure 1 for a visual diagram of the diagnostic results. A good topic solution should optimally maximize both exclusivity and semantic coherence, which are in tension with each other. Potential topic solutions can be identified by point values relatively closer to the top left corner of the figure. (To illustrate, in the included figure a seven-topic model unequivocally outperforms a five-topic model.) The diagnostic results suggested four potential solutions (6, 7, 10, and 14 topics) as outperforming the remainder. We estimated each of these topic-number models and analysed the resulting topics qualitatively and eliminated the 10 and 14 topic solutions for poor interpretability. We compared the six- and seven-topic solutions more fulsomely, and eventually eliminated the seven-topic solution in favour of the more qualitatively meaningful six-topic model. Consequently, we proceeded with the six-topic solution.

The highest probability terms for each of the six topics are listed in Table 1, using four metrics for topic membership. According to the model, these words have the highest probability of belonging to the topic when they appear within the text. Additional information on the nature and calculation of each is beyond the scope of this manuscript and we refer readers to the technical literature [see (36)]. To summarize, *Highest* refers to the words with the highest probability of belonging to the topic (43). *FREX* and *Lift* reduce the probability for words that are shared amongst multiple topics, identifying the words with greater exclusivity to the topic (43). *Score* adjusts for overall word frequency, pinpointing less commonly used terms (43). We include all metrics here for thoroughness and transparency.

**Table 1:** Associated words per topic for six-topic model.

Topic number	Words with highest probability of belonging to topic	Initial interpretation by data analyst
1	Highest: need, servic, disabl, child, privat, peopl, involv	Appropriately meeting all needs
	FREX: disabl, privat, public, therapi, etc, evalu, spectrum	
	Lift: cost, defin, embodi, govern, harm, ignor, injustic	
	Score: disabl, etc, evalu, harm, injustic, righteous, midst	
2	Highest: feel, week, languag, teacher, communic, team, need	Teamwork, collaboration, and partnership within the school
	FREX: week, feel, part, sens, team, target, growth	
	Lift: partner, valuabl, accomplish, faster, husband, incorpor, most	
	Score: accomplish, week, incorpor, member, real, partner, connect	
3	Highest: tier, student, teacher, educ, program, strategi, classroom	Developing capacities within the classroom
	FREX: tier, strategi, referr, feedback, may, two, play	
	Lift: check-in, guest, essenti, grammat, potenti, prior, specialti	
	Score: tier, narrat, feedback, student, indic, strategi, potenti	
4	Highest: student, languag, speech, need, classroom, servic, back	Supporting individual student needs within the classroom
	FREX: languag, pathologist, back, slps, speech, student, build	
	Lift: graduat, path, pronoun, advic, anxieti, bodi, built	
	Score: student, stutter, languag, impact, intervent, cdas, confid	
5	Highest: communic, child, slp, speech, need, support, children	Coordinating services and supports for children with greater needs
	FREX: child, devic, train, name, attend, region, slp	
	Lift: anxious, design, dress, fact, fulli, googl, offici	
	Score: child, arrang, statist, pec, surpris, devic, except	
6	Highest: teacher, student, read, impact, want, decod, support	Supporting core educational skills and goals
	FREX: decod, phonem, read, level, term, awar, instruct	
	Lift: equip, instanc, product, advanc, bang, buck, checklist	
	Score: decod, phonem, impact, benchmark, reader, instruct, three	

**Figure 2:**
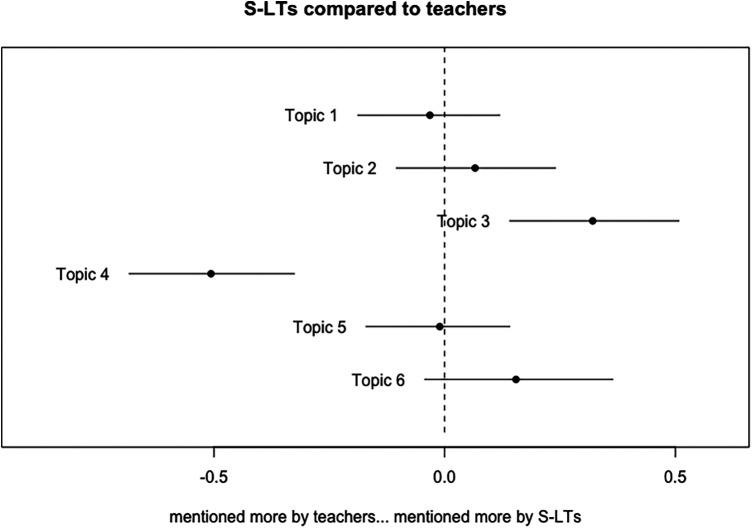
S-LT topic proportion differences compared to teachers with point estimates and 95% confidence intervals.

**Figure 3:**
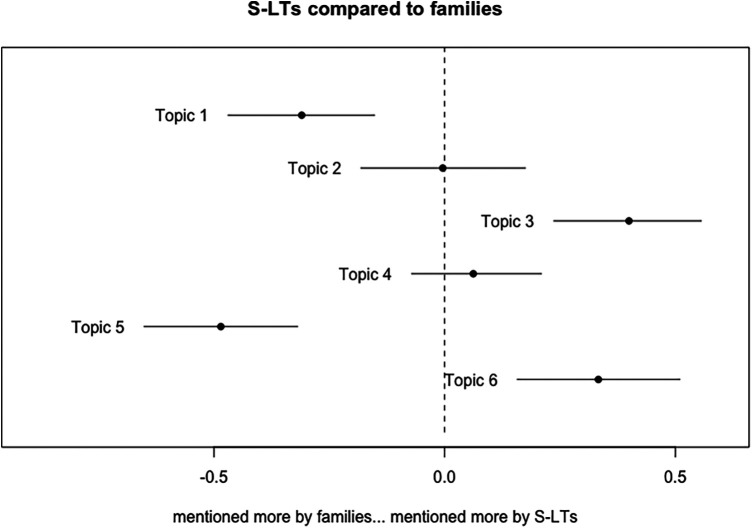
S-LT topic proportion differences compared to families with point estimates and 95% confidence intervals.

We then estimated the prevalence of each topic within text from each participant group. As this work is situated within the disciplinary perspective of speech and language therapy, we used the S-LTs as the reference group for comparison. In this way, we would be able to identify topics that teachers and families discussed significantly more or less when compared to S-LTs, suggesting potential divergences in group members’ perspectives. Figures 2, 3 present the point estimates and 95% confidence intervals for topic proportions across participant groups. In both cases, positive values indicate that S-LTs discussed the topic more, whereas negative values indicate that the comparison group (educators and families) discussed the topic more. Zero (indicated in the figures with the dotted vertical line) signals that the data are consistent with no differences in topic proportions between groups. Compared to teachers, S-LTs discussed topic 3 more and topic 4 less. S-LTs may have also dedicated more attention to topic 6, although the data are also consistent with no difference. Topics 1, 2, and 5 did not vary in proportions between S-LTs and teachers.

Compared to families, S-LTs discussed topics 3 and 6 more, and 1 and 5 less. The data were consistent with no differences in prevalence for topics 2 and 4. Specific values for coefficients, standard errors, *t*- and *p*-values can be found in the Additional file 2.

### 3.3 Step 3. Qualitative interpretation and categorization

After completing data familiarization and structural topic modelling, we then qualitatively interpreted both previous steps. Greater detail regarding the quality of what was said relevant to each topic is provided below, along with emblematic quotes.

### 3.4 Topic 1—appropriately meeting all needs

The content within the topic focused on meeting all needs within the school. Family members discussed this topic more than S-LTs and indicated that sufficient supports were not available within the school system to adequately need the needs of all students. For example, one parent stated:

*“When you have these two people servicing a few individuals who need it, it shows you need so much more in order to service all these other kids that really do not need as much care and attention… But right now, it seems like it’s just, this is what we are picking [the children receiving intensive services]. This is what all we have and that’s who gets it and that’s it. So, what about everybody else?” Family member 7*

Family members indicated that those families who could frequently turned to private speech-language services outside of the schools to meet the needs of their children, while recognizing that this was problematic and inequitable to many families. One family member reported frustration with consistently needing to access resources outside of the school, and the negative impacts the family was suffering as a result.

*“I had to go through other side channels and try to get either information or like any kind of like, you know, to push things forward. Like I said, even [child’s name] being transferred to a completely different platform, educational platform, has never been offered to me, or presented as an option to me by the school… She will be starting grade one, and she’s not going to be on the educational plan for grade one, which is a complete disaster.” Family member 3*

S-LTs and educators also expressed concern regarding meeting all needs within the school and noted the substantial staffing and resource challenges within their workplaces, albeit less frequently and forcefully compared to the family members. One S-LT suggested that there was great uncertainty in how to best allocate resources to meet needs, and that this was a major barrier to offering impactful services in schools.

*“I think that having more information about the things that are impactful would be beneficial in terms of prioritizing the caseload and managing the caseload. Absolutely. You know, there, there are times when you spend a lot of time with it with a student, and the educators, and the assistants, and the parents, but in the end, you really do not know the impact that you are having. You just feel that well this is what I should be doing this is how I think it would help.” S-LT 13*

### 3.5 Topic 2—teamwork, collaboration, and partnership within the school

The content of this topic focused on the importance of teamwork, collaboration, and partnership within the school. All participants discussed this topic at length. S-LTs and educators frequently emphasized the critical role that collaboration held within school-based practice. For example, one teacher stated:

*“That is the most integral part of educating the student. And so, when we are just with me and my educational partners my teaching partners, it is the co-teaching, co-assessing. But then, with all of our outside support services like S-LP [S-LT], and the community services. You have to have the mindset that nobody knows more than the other but that it is like a symbiotic relationship where I am going to learn from you, and you are going to learn from me. And we kind of have that time and space to work together. It has been impactful and in my experience. I have always been open to anybody who is going to help me bring my students forward.” Educator 1*

Family members discussed wanting to be more involved with the school team, and for more open and consistent communication with the S-LTs and educators. A desire for a more proactive and engaging approach from the school was also reported by family members. For example, one participant stated the following.

*“It should not be me to be the expert. Even though I am not, I felt like I became one. It is supposed to be them who will be teaching and guiding me instead of me trying to figure out how to arrange a training for certain number of people, so that they will know how to support my child’s needs while she is there, and I told them that I really want us to work as a team. I do not want the burden to be on you only but at the same time you have to do something from your side.” Family member 3*

### 3.6 Topic 3—developing capacities within the classroom

The content of this topic was focused on how S-LTs could support teachers, educational assistants, and other professionals working in the classroom, building their capacities to support their students’ needs. S-LTs discussed this topic more when compared to both educators and families and building staff capacity seemed to be considered a core aspect of achieving desired outcomes within school-based practice.

*“For me it truly feels that when I'm able to educate the teacher around what they can do in the-every-day. I am only there once a week, most of the time. So once, once they start implementing the strategies that I give every single day, they know. They notice a difference. They notice an impact.” S-LT 5*

Building staff capacity included both the skills and knowledge of teachers and other school personnel, as well as their confidence and positive attitude towards supporting children with communication difficulties within the classroom.

*“There are many people who feel like, if they have a student, that they are struggling with. When I say struggling with, I mean feeling like they are not making a strong effect on and not being able to teach them and move them along. Then the feeling is, they want someone else to come in and help them. And what we really want to do is we really, really, really want to provide teachers, educators with the feeling that they have the skills.” S-LT 12*

When family members discussed this topic, they included everyone within the school as benefiting from capacity and knowledge development. For example, one parent suggested that the S-LT spend time in the classroom educating peers about communication disorders and inclusive practices.

*“To me, the important thing is trying to make it inclusive for the child. So, if the S-LP [S-LT] is going to come into the class, then I think it would be a great idea for them to say hey guys you know I am the speech therapist. And this is to the whole class not to my child only, to say I am a speech therapist and there is some children who sometimes have difficulty with language, with communication, with all these different things, and I am here to help. And these are some of the things that we can do.” Family member 9*

### 3.7 Topic 4—meeting specific student needs within the classroom

The content of the fourth topic focused on how to support specific students within the classroom. Educators discussed the topic more than did S-LTs. Teachers emphasized the need for supports, strategies, and suggestions to make sense within the educational context. One educator emphasized how having school-based S-LTs as opposed to external professionals helped ensure impactful recommendations to support children within their educational context.

*“And I think by having speech and language in the buildings, it is helping to close that gap significantly. Because especially with special education, a lot of times we have outside providers that will come in, and in the past this has been speech and language, that will make recommendations and say, you know what you can just do this, and you can do this, and you can do this, which is all great in theory and in a supervised setting or a one-on-one setting or a nice, quiet environment, it is ideal. But when you bring that into the regular chaos of the classroom, and all the other needs that are in there, it is not always applicable. And I think by having speech and language in the building, they are seeing now more what is happening in the classroom environment, and then they are adapting the programming and the services to meet to better meet those needs. And I think that has helped immensely as well.” Educator 10*

Educators also reported an appreciation for the speciality skills brought into the classroom by S-LTs, and how these skills could be leveraged into specific daily practices.

*“They [S-LTs] are often the ones that are able to pinpoint the specific need that a child has. So, when I’m working with a student and I know that there is gaps in their language, or their speech, I might be able to take a guess at what areas they need to develop… But because I do not have that trained ear that you guys have when you are doing an assessment, I am really just guessing. I am guessing at what sounds are missing. And oftentimes the speech language pathologist [S-LT], they will come back, and they will be very specific and say, oh, you know what, in language, it is actually their word retrieval, or it is their sounds that they make with “tr” or something that. So, they are very specific. And then when they work with the children, they are able to give me specific ways that I can help the child improve with their language and their speech on a daily basis.” Educator 11*

### 3.8 Topic 5—coordinating services and supports for children with greater needs

The content of this topic concentrated on care coordination to support individual student needs and was a major focus for family members. Families expressed a strong preference for care coordination within schools and reported negative feelings about the effort required to advocate for care coordination for their children. For example, one parent stated:

*“I am expecting that that support and that implementation will be in place before even I reach out. Not once I put foot in that school and then, they are going to start to search. Okay, whom do we need? Like you cannot gather a team or try to figure out, okay, what do we need to support this child? So, you should have some sort of a process and people in place already available so that a child like mine comes in, they will know what to do from day one.” Family member 6*

S-LTs being responsive to children's holistic needs also was mentioned frequently. Educators noted that S-LTs were frequently the point of entry for other referrals, such as to formal assessment for social communication challenges. Parents reported valuing S-LTs proactively coordinating or initiating interprofessional collaboration to support the child as a whole person.

*“And then the other thing is just having that view of the child that I am going to look at a child was a whole person. And okay I am supposed to focus on his speech, but is there anything else that might be hindering him from being successful? So, if you know if you can see that my child you know cannot regulate himself or their sensory needs, you know, then you know to me the S-LP [S-LT] then should within their school team say, you know what, in my, in my sessions I am finding that you know he cannot really concentrate. He sort of looks like he needs to have a lot of movement. Or I see that he is struggling a lot with fine motor. So can we refer him for OT [occupational therapy] services, you know, so to me that is looking at the whole child or, you know, her saying, you know mom is coming to me and saying, you know, he cannot even toilet himself. So do we have supports in place for that?” Family member 9*

Compared to family members, educators reported most positively about care coordination within schools and emphasized how S-LTs had impacted the ability of the system to respond rapidly to referrals. Teachers also emphasized that this care coordination is effective when conducted within the school, and that they would not expect the same outcomes from S-LTs sent from external agencies.

*“Really the biggest change for any support for any kid anywhere is waitlist. I think we do a pretty good job in our [school] board though with, like, I have to say our speech and language team has been right on top of everything this year and getting in and assessing kids. We are able to start to put programming in place pretty quickly. Outside supports, there is, you know, if we have to send a kid to school-based support [provided by an external agency], then that is like a yearlong waitlist and then they only come in a few times, maybe 10 times a year, to see the student.” Educator 6*

### 3.9 Topic 6—supporting core educational skills and goals

The content of the final topic focused on how S-LTs could support core educational skills and goals, with a particular focus on literacy instruction. S-LTs discussed how they felt that they could support teachers in evidence-based practices relevant to core educational skills, and provide material resources, training, and other supports to improve educational practices. For example, one S-LT reported highly valuing this outcome.

*“I just really want to have more of an impact in supporting literacy development within the schools because it is a little bit disorganized right now within our school system. There is very inconsistent access to literacy supports from one school to the next, and I find that that’s where a lot of the educators are coming to me for support, and we do not have the time to give as much support as I would like to. So, my biggest impact that I want to make is continuing to empower and enable educators to enhance their literacy skills and their literacy support for students.” S-LT 5*

Supporting children's educational journeys was also reported to be a core aspect of speech-language practice in schools according to the S-LTs, and that this aspect of practice was unique to working within a school-based context. One S-LT highlighted how they considered students’ educational success as the most distal outcome of services in schools, and how practice must be oriented towards achieving this success.

*“Ultimately, like I said, the goal is having them in the classroom and supporting them in the classroom. So, in terms of how successful they are in the classroom that is then, I believe, kind of an indirect reflection of how successful they are with those strategies and supports that we have recommended, and those strategies and supports are then helping them to access curriculum and to be successful in the classroom, which is our ultimate goal.” S-LT 4*

Educators also discussed the importance of keeping the child in the classroom accessing core educational activities, and that S-LTs providing these supports could help educators achieve their desired educational outcomes more effectively and efficiently.

*“Tier one is how the S-LP [S-LT]… is supporting the classroom teacher. So how are you supporting them so that they can deliver better material and better lessons and so on. So you are guiding their practice, as opposed to being the one to kind of directly do it… they could talk about those strategies about what we do and why we do it how it is helpful and how those spelling tests you have done every week, you know, they did have a purpose but now we can focus on this because we want to get more bang for our buck. We want to make sure that the time we are spending on these areas with kids is actually more effective.” Educator 2*

Parents discussed this topic less frequently compared to S-LTs yet indicated sentiments consistent with the outcomes the S-LTs reported as valuing, such as maintaining students within an inclusive classroom with their peers, learning with and from their classmates. However, family members connected this outcome to topic 5 (care coordination), rather than the supports to core educational skills and goals, which was highlighted by S-LTs.

### 3.10 Overarching issues related to outcomes

Some participants proffered perspectives on the use of outcomes in school-based practice. Multiple participants pointed out inconsistencies or challenges with indicators (specific measures for an outcome). For example, one educator reported that what was measurable was not what mattered, and that important outcomes required qualitative assessment rather than measurement.

*“I need to see you know benefits in their day-to-day life that maybe are not the most measurable things but are more important. It is interesting to see like if they are collecting data in like certain ways. But I do not think everything that is always the most important thing that we, as teachers, or as parents, are looking for are always the most measurable things. They are maybe something that can be reflected on more anecdotally.” Educator 11*

In contrast, a parent reported similar dissatisfaction with current measurement techniques, yet emphasized the need for a quantitative approach.

*“We want to see growth, right? But how do we measure that growth? I think that is key. Like if there was some sort of assessment, or where it is streamlined, so that everyone is using it and that information is shared. Like it is hard to see growth unless it is, I don't know, numbers based, or if it is quantitative data, I guess you would say. Data that is actually real.” Family member 8*

S-LTs also reported frustration with their current ability to assess and make judgements about the outcomes of their services, and that further work in this area was important for the development of the profession.

*“I guess just in general I mean I think we have a lot of impact in the schools, but they are just not just really not recognized, I think. We really do not. There is not a really objective way for us to know what the impacts are.” S-LT 13*

All participant groups reported that the measurement or qualitative assessment of important outcomes would contribute to improving school-based services, and there was general agreement that current measurement techniques are not sufficiently developed to provide robust, meaningful information about the impact of practice within schools.

## 4 Discussion

In this study, we interviewed S-LTs, educators, and family members about their perceptions of meaningful outcomes for school-based speech-language therapy services. After initial qualitative reading of all data, structural topic modelling was used to identify six latent topics within the interview data, and the quality of the content within each topic was explored through further qualitative analysis. The results are broadly consistent with previous literature, confirming important areas for further work on outcomes in the discipline. However, they provide additional nuance and detail.

Consistent with previous literature (18, 20, 33), the participants in this study considered multiple outcomes beyond individual student clinical outcomes to be important, including outcomes related to partnership and collaboration as well as system-functioning. Additionally, it was evident that these partnership and systems outcomes were valued across participant groups, with S-LTs emphasizing collaboration and capacity building with the school team for example, and family members discussing the importance of coordinated care that was responsive to all needs. Such outcomes have been noted to be infrequently included in SLT research to date (10), and the implementation of new outcomes in research and practice remains an important area for future growth within the profession. These results reinforce calls from the limited previous literature (18, 20, 33) on this topic for research in the profession to expand dramatically beyond its traditional clinical outcomes, considering a broader scope of outcomes more consistent with a biopsychosocial approach to health. Without considering these partnership and collaboration outcomes, research in the area will be unable to provide evidence-based guidance to inform the most meaningful decisions for these important services.

Similar to the work done by Gallagher and colleagues in Ireland (17), we spoke with family members, educators, and S-LTs, with similar topics present in our discussions with participants. For example, the participants in our study also spoke to the value of children participating meaningfully in the academic and social life of schools, as well as understanding how to engage with learning activities and their peers. Participants also mentioned children implementing new skills to be more independent and successful in the classroom as an important outcome. These sentiments all closely reflect the previous findings (17). Maximizing the time students spend in the classroom with learning and interacting with their peers also was endorsed by all participant groups in this study, reflecting the previously reported desire of children with communication to remain in inclusive environments and not to be labelled and separated from their classmates (11–13). Therefore, an increase in the time the children spend within the classroom or a reduction in the time spent withdrawing the student for supports may be an important outcome of service delivery in schools. Our results also are consistent with previous work suggesting that proactive communication and care coordination with families was an important desired outcome of rehabilitation services in schools (15). Ng et al.'s (15) ethnographic study was conducted in the same province where our study was completed, suggesting that care coordination may be an important outcome in this particular context. Finally, our results are consistent with the observation by Murphy (34) that the outcomes valued most by school community members are not frequently included in research. The outcomes measured in studies of school-based service delivery to date [see (44, 45)] have been narrowly defined clinical outcomes, such as standardized test scores and specific trained skill and generalization probes. These types of outcomes, although important, do not reflect all relevant aspects of service impact and care quality. The continued exclusion from research studies of outcomes that families, educators, and S-LTs deem meaningful will likely reduce the relevance of the evidence base for informing practice. Based on previous studies, S-LTs working in schools have innovated around this limitation in the research, finding new ways to measure and evaluate the impact of their services (46), although they report the need for additional support to continue to develop and innovate. An expanded and improved research base may be of great utility in fostering further innovation in practice.

Inconsistent with previous work, we did not observe a substantive focus on the children's voice directing or informing the supports they receive in schools, something which has been found in other studies (17, 47). This is likely because we did not speak directly with children with disabilities, something that was a focus of these previous studies (17, 47). The content of topic one was unexpected, as family members discussed the importance of providing sufficient supports to all children in schools as a public good, and that families turning to the private sector for services was considered an indicator of unsuccessful service delivery models within schools. It is unclear if this finding primarily reflects the context in which our research was conducted. Finally, we note that previous work (18) in this locale has identified accountability to systems as an outcome that drives decision-making, where demonstrating to managers, regulatory bodies, or funders that certain types or frequencies of services are being provided, or that certain standards are being met are an important part of determining the outcomes of services in schools. In that study, we asked experienced clinicians and clinical managers to describe what outcomes were used in their schools and local education authorities. In the present study, we asked multiple groups from school communities about the outcomes that they valued, and accountability to systems was present in the data, suggesting that such outcomes, although they may be required in certain organizational contexts, are not informative regarding whether S-LT services in schools are truly achieving valued outcomes.

In summary, this study confirmed that multiple types of outcomes, including those relevant to individual students, partnership and collaboration in schools, care coordination, and capacity building (among others) were considered valuable or important outcomes by family members, educators, and S-LTs. These topics were present in the data from all participants, suggesting that they may all be important outcomes of S-LT services in schools. However, there were difference among participants regarding the quantity they discussed each. S-LTs focused more than the other school community members on capacity building and supporting core educational skills and goals; family members focused on meeting the needs of all students and providing responsive and well-coordinated care; finally, educators focused on problem solving and strategy implementation to support individual students. These differences in emphasis by various members of the school community should be explored further in future work, and a consensus exercise to identify the most important core outcomes of SLT services in schools may prove fruitful.

### 4.1 Limitations

This study has several limitations. First, although we included multiple groups from the school community who have a vested interest in school-based services, we did not include one very critical member group of this community. We did not speak directly with children. Although children appear to agree with their parents, teachers, and S-LTs regarding what outcomes they value, children also bring a nuanced interpretation of the same (17). We hope to explore what these outcomes mean to children who receive such services in future work. Additionally, we recruited participants only from a narrow geographical area. This design choice potentially limited the diversity of included perspectives by excluding those who did not reside within a specific locale, which may suggest additional outcomes as relevant to tiered, school-based services beyond those which we identified.

Further, this study has important theoretical limitations. We approached the issue of outcomes with the assumption that quantifying outcomes of services is a meaningful method for evaluating service quality. In previous work (18), clinicians have questioned this assumption regarding the primacy of outcome quantification over rich, narrative information on student and system functioning. Interestingly, some participants who contributed to the present study also questioned this approach. Had we grounded our analysis in other paradigmatic perspectives, we may have arrived at different results about the roles of outcomes in health service delivery and evaluation. Such perspectives may be valuable to promote reflexivity and growth within the profession of speech-language therapy.

## 5 Conclusion

In this study, we asked family members, educators, and clinicians about the most important and valued outcomes of speech-language therapy services delivered in schools. Structural topic modelling revealed six broad outcome concepts identified as important by these stakeholder participants. These outcome concepts included: meeting the needs of all students; teamwork, collaboration, and partnerships within the school; building capacities within the classroom to support student needs; supporting individual student needs within the classroom; coordinating services and supports for students with greater needs; and, finally, supporting core educational skills and goals. Although all outcome concepts were discussed by all participants, there were several differences among S-LTs relative to educators and family members regarding the quantity of data dedicated to each, suggesting differences in how different members of the school community valued each outcome concept. The outcomes identified as important were notably neither those included in research to date, nor were they considered feasibly measured with current outcome measures and assessment tools. To further build from this work, we recommend consensus and prioritization work to identify the core outcomes for school-based service delivery and the most urgent outcome measure development and implementation for school-based services.

## Data Availability

The raw data supporting the conclusions of this article will be made available by the authors, without undue reservation.

## References

[1] Donabedian A. The role of outcomes in quality assessment and assurance. Qual Rev Bull. (1992) 18(11):356–60. 10.1016/S0097-5990(16)30560-7 1465293

[2] Donabedian A. Evaluating the quality of medical care. Milbank Q. (2005) 83(4):691–729. 10.1111/j.1468-0009.2005.00397.x 16279964 PMC2690293

[3] Santana MJ Manalili K Jolley RJ Zelinsky S Quan H Lu M. How to practice person-centred care: a conceptual framework. Heal Expect. (2018) 21(2):429–40. 10.1111/hex.12640 PMC5867327 29151269

[4] Kuo DZ Houtrow AJ Arango P Kuhlthau KA Simmons JM Neff JM. Family-centered care: current applications and future directions in pediatric health care. Matern Child Health J. (2012) 16(2):297–305. 10.1007/s10995-011-0751-7 21318293 PMC3262132

[5] Mühlbacher AC Juhnke C. Patient preferences versus physicians’ judgement: does it make a difference in healthcare decision making? Appl Health Econ Health Policy. (2013) 11(3):163–80. 10.1007/s40258-013-0023-3 23529716

[6] Laver K Ratcliffe J George S Lester L Crotty M. Preferences for rehabilitation service delivery: a comparison of the views of patients, occupational therapists and other rehabilitation clinicians using a discrete choice experiment. Aust J Occup Ther. (2013) 60(2):93–100. 10.1111/1440-1630.12018 23551002

[7] Raymond MH Demers L Feldman DE. Differences in waiting list prioritization preferences of occupational therapists, elderly people, and persons with disabilities: a discrete choice experiment. Arch Phys Med Rehabil. (2018) 99:35–42. 10.1016/j.apmr.2017.06.031 28797617

[8] Barratt A. Evidence based medicine and shared decision making: the challenge of getting both evidence and preferences into health care. Patient Educ Couns. (2008) 73(3):407–12. 10.1016/j.pec.2008.07.054 18845414

[9] Cunningham BJ Washington KN Binns A Rolfe K Robertson B Rosenbaum P. Current methods of evaluating speech-language outcomes for preschoolers with communication disorders: a scoping review using the ICF-CY. J Speech Lang Hear Res. (2017) 60(February):446–64. 10.1044/2016_JSLHR-L-15-0329 28219081

[10] Baker E Masso S Huynh K Sugden E. Optimizing outcomes for children with phonological impairment: a systematic search and review of outcome and experience measures reported in intervention research. Lang Speech Hear Serv Sch. (2022) 53(July):732–48. 10.1044/2022_LSHSS-21-00132 35394819

[11] Markham C Van Laar D Gibbard D Dean T. Children with speech, language and communication needs their perceptions of their quality of life. Int J Lang Commun Disord. (2009) 44(5):748–68. 10.1080/13682820802359892 19107658

[12] Lyons R Roulstone S. Labels, identity and narratives in children with primary speech and language impairments. Int J Speech Lang Pathol. (2017) 19(5):503–18. 10.1080/17549507.2016.1221455 27631150

[13] Lyons R Roulstone S. Well-being and resilience in children with speech and language disorders.. J Speech, Lang Hear Res. (2018) 61(2):324–44. 10.1044/2017_JSLHR-L-16-0391 29374284

[14] Marshall J Harding S Roulstone S. Language development, delay and intervention—the views of parents from communities that speech and language therapy managers in England consider to be under-served. Int J Lang Commun Disord. (2017) 52(4):489–500. 10.1111/1460-6984.12288 27995697

[15] Ng SL Lingard L Hibbert K Regan S Phelan S Stooke R Supporting children with disabilities at school: implications for the advocate role in professional practice and education. Disabil Rehabil. (2015) 37(24):2282–90. 10.3109/09638288.2015.1021021 25738906 PMC4673542

[16] Kwok E Bootsma J Cahill PT Rosenbaum P. A scoping review of qualitative studies on parents’ perspectives on speech, language, and communication interventions. Disabil Rehabil. (2021) 44(25):8084–809. 10.1080/09638288.2021.1989061 34669539

[17] Gallagher AL Murphy C Conway PF Perry A. Engaging multiple stakeholders to improve speech and language therapy services in schools: an appreciative inquiry-based study. BMC Health Serv Res. (2019) 19(26). 10.1186/s12913-019-4051-z PMC6466713 30987610

[18] Cahill PT Ng SL Dix L Ferro MA Turkstra LS Campbell WN. Outcomes management practices in tiered school-based speech-language therapy: a Canadian example. Int J Lang Commun Disord. (2022) 58(3):786–801. 10.1111/1460-6984.12822 36426768

[19] Blosser J. Outcomes matter in school service delivery. In: Frattali CM Golper LAC, editors. Outcomes in Speech-Language Pathology. 2nd ed. New York, NY: Thieme Medical Publishers, Inc (2013). p. 116–40.

[20] VanderKaay S Dix L Rivard L Missiuna C Ng S Pollock N Tiered approaches to rehabilitation services in education settings: towards developing an explanatory programme theory. Int J Disabil Dev Educ. (2021. 10.1080/1034912X.2021.1895975

[21] Hsieh HF Shannon SE. Three approaches to qualitative content analysis. Qual Health Res. (2005) 15(9):1277–88. 10.1177/1049732305276687 16204405

[22] Eickhoff M Wieneke R. Understanding topic models in context: a mixed-methods approach to the meaningful analysis of large document collections. Proc 51st Annu Hawaii Int Conf Syst Sci (2018). p. 903–12

[23] Gentles SJ Vilches SL. Calling for a shared understanding of sampling terminology in qualitative research: proposed clarifications derived from critical analysis of a methods overview by McCrae and purssell. Int J Qual Methods. (2017) 16(1):1–7. 10.1177/1609406917725678

[24] Gentles SJ Charles C Ploeg J Ann McKibbon K. Sampling in qualitative research: insights from an overview of the methods literature. Qual Rep. (2015) 20(11):1772–89. 10.46743/2160-3715/2015.2373

[25] Macharis C Turcksin L Lebeau K. Multi actor multi criteria analysis (MAMCA) as a tool to support sustainable decisions: state of use. Decis Support Syst. (2012) 54(1):610–20. 10.1016/j.dss.2012.08.008

[26] Banville C Landry M Martel J-M Boulaire C. A stakeholder approach to MCDA. Syst Res Behav Sci. (1998) 15:15–32. 10.1002/(SICI)1099-1743(199801/02)15:1<15::AID-SRES179>3.0.CO;2-B

[27] Malterud K Siersma VD Guassora AD. Sample size in qualitative interview studies: guided by information power. Qual Health Res. (2016) 26(13):1753–60. 10.1177/1049732315617444 26613970

[28] Creswell JW Hirose M. Mixed methods and survey research in family medicine and community health. Fam Med Community Heal. (2019) 7(2):1–6. 10.1136/fmch-2018-000086 PMC6910743 32148709

[29] Harris PA Taylor R Minor BL Elliott V Fernandez M O’Neal L The REDCap consortium: building an international community of software platform partners. J Biomed Inform. (2019) 95(103208). 10.1016/j.jbi.2019.103208 31078660 PMC7254481

[30] Elo S Kyngäs H. The qualitative content analysis process. J Adv Nurs. (2008) 62(1):107–15. 10.1111/j.1365-2648.2007.04569.x 18352969

[31] Isoaho K Gritsenko D Mäkelä E. Topic modeling and text analysis for qualitative policy research. Policy Stud J. (2021) 49(1):300–24. 10.1111/psj.12343

[32] R Core Team. R: A language and environment for statistical computing. Vienna, Austria: R Foundation for Statistical Computing (2021).

[33] Terreberry S Dix L Cahill PT Passaretti B Campbell WN. Moving towards a tiered model of speech and language services in Ontario schools: perspectives of school-board speech-language pathologists. Can J Speech-Language Pathol Audiol. (2021) 45(4):267–82.

[34] Murphy CA. The limits of evidence and the implications of context: considerations when implementing pathways to intervention for children with language disorders. Int J Lang Commun Disord. (2019) 54(1):20–3. 10.1111/1460-6984.12425 30565802

[35] Roberts ME Stewart BM. Tingley D. Stm: an R package for structural topic models. J Stat Softw. (2019) 91(2):1–40. 10.18637/jss.v091.i02

[36] Roberts ME Stewart BM Tingley D Lucas C Leder-Luis J Gadarian SK Structural topic models for open-ended survey responses. Am J Pol Sci. (2014) 58:1064–82. 10.1111/ajps.12103

[37] Lucas C Nielsen RA Roberts ME Stewart BM Storer A Tingley D. Computer-assisted text analysis for comparative politics. Polit Anal. (2015) 23(2):254–77. 10.1093/pan/mpu019

[38] Mimno D Wallach HM Talley E Leenders M McCallum A. Optimizing semantic coherence in topic models. Proc 2011 Conf Empir Methods Nat Lang Process Proc Conf (2011). p. 262–72

[39] Roberts ME Stewart BM Airoldi EM. A model of text for experimentation in the social sciences. J Am Stat Assoc. (2016) 111(515):988–1003. 10.1080/01621459.2016.1141684

[40] Teddlie C Tashakkori A. Foundations of Mixed Methods Research: Integrating Quantitative and Qualitative Approaches in the Social and Behavioural Sciences. Thousand Oaks, CA: SAGE Publications, Inc. (2009).

[41] Onwuegbuzie AJ Johnson RB Collins KMT. Assessing legitimation in mixed research: a new framework. Qual Quant. (2011) 45(6):1253–71. 10.1007/s11135-009-9289-9

[42] Albalawi R Yeap TH Benyoucef M. Using topic modeling methods for short-text data: a comparative analysis. Front Artif Intell. (2020) 3(00042). 10.3389/frai.2020.00042 33733159 PMC7861298

[43] Kuo I-C Huang W. Does title or content matter?: examining China’s partnerships with text classification. In: Wei W, editors. China’s Contemporary Image and Rhetoric Practice. London, UK: Routledge (2021). p. 3–29.

[44] Archibald LM. SLP-educator classroom collaboration: a review to inform reason-based practice. Autism Dev Lang Impair. (2017) 2:1–17. 10.1177/2396941516680369

[45] Cirrin FM Schooling TL Nelson NW Diehl SF Perry FF Staskowski M Evidence-based systematic review: effects of different service delivery models on communication outcomes for elementary school-age children. Lang Speech Hear Serv Sch. (2010) 41:233–64. 10.1044/0161-1461(2009/08-0128) 20421615

[46] Cahill PT Ng S Dix L Ferro MA Turkstra L Campbell WN. Outcomes management practices in tiered school-based speech–language therapy: a Canadian example. Int J Lang Commun Disord. (2022) 58:786–801. 10.1111/1460-6984.12822 36426768

[47] Paul T Di RB Rosenbaum P Cahill PT Jiang A Kim E Perspectives of children and youth with disabilities and special needs regarding their experiences in inclusive education: a meta-aggregative review. Front Eduction. (2022) 7(864752).

